# A Two-Year Evaluation of Corrosion-Induced Damage to Hot Galvanized Reinforcing Steel B500SP in Chloride Contaminated Concrete

**DOI:** 10.3390/ma13153315

**Published:** 2020-07-25

**Authors:** Mariusz Jaśniok, Maria Sozańska, Jacek Kołodziej, Bartosz Chmiela

**Affiliations:** 1 Faculty of Civil Engineering, Silesian University of Technology, 5 Akademicka, 44-100 Gliwice, Poland; jacek.kolodziej@polsl.pl; 2 Faculty of Materials Engineering, Silesian University of Technology, 8 Krasińskiego, 40-019 Katowice, Poland; maria.sozanska@polsl.pl (M.S.); bartosz.chmiela@polsl.pl (B.C.)

**Keywords:** concrete, reinforcing steel, zinc coating, HDG, corrosion, chlorides, LPR, EIS, SEM, EDS

## Abstract

Corrosion-induced damage to concrete reinforced with bars is a serious problem regarding technical and economic aspects and strongly depends on used materials, corrosion environment, and service life. Tests described in this paper refer to a two-year evaluation of the effectiveness of protection provided by zinc-coated low-carbon reinforcing steel of grade B500SP in concrete against chloride corrosion. Performed tests were comparative and included measurements conducted on four groups of concrete test elements with dimensions of 40 mm × 40 mm × 140 mm reinforced with a bar having a diameter of ϕ8 mm. Particular groups were a combination of different types of concrete with or without chloride additives, with galvanized or black steel. Chlorides as CaCl_2_ were added to the concrete mix in the amount of 3% of cement weight in concrete. Reinforced concrete specimens were periodically monitored within two years using the following techniques: linear polarization resistance (LPR) and electrochemical impedance spectroscopy (EIS). Polarization measurements were conducted in a three-electrode arrangement, in which a rebar in concrete served as a working electrode, stainless steel sheet was used as an auxiliary electrode, and Cl^−^/AgCl,Ag was a reference electrode. Comparative tests of changes in the density of corrosion current in concrete specimens without chloride additives basically demonstrated no development of corrosion, and possible passivation was expected in case of black steel. Higher densities of corrosion current were observed for galvanized steel during first days of testing. The reason was the dissolution of zinc after the contact with initially high pH of concrete pore solution. Six-month measurements demonstrated a higher density of corrosion current in concrete specimens with high concentration of chlorides, which unambiguously indicated corrosion in concrete reinforced with galvanized or black steel. Densities of corrosion current determined for selected specimens dramatically decreased after an 18-month interval in measurements. Corrosion was even inhibited on black steel as an insulating barrier of corrosion products was formed. The above observations were confirmed with structural studies using scanning electron microscopy (SEM) and energy dispersive spectroscopy (EDS) techniques. Results obtained from corrosion (LPR, EIS) and structural (SEM, EDS) tests on specimens of concrete reinforced with steel B500SP demonstrated a very favorable impact of zinc coating on steel by providing two-year protection against corrosion in the environment with very high chloride content.

## 1. Introduction

Issues addressed to the durability of reinforced concrete structures have been much tested and described in scientific papers of an interdisciplinary nature [1,2]. Bonds formed between concrete and steel reinforcement are the main used specificity of reinforced concrete as the construction material [3,4]. Therefore, concrete in a building transmits main compressive stresses, and steel transmits tensile stresses [5]. Regarding durability in the corrosion environment, reinforced concrete should be identified with a steel electrode (half-cell) immersed in the highly alkaline pore solution filling the porous structure of concrete [6,7].

A combination of two different construction materials, being advantageous from the mechanical point of view, can create serious difficulties and complications related to the evaluation of the structure safety with reference to its durability [8,9,10]. The main problem is the lack of direct access to steel reinforcement covered with concrete or failure to make observations without measuring equipment. Carbon dioxide inducing concrete carbonation [7,11] and chlorides [12] present in, inter alia, deicing agents or saltwater are among the most serious and common factors creating corrosion risk to reinforcement. Mechanisms resulting in damage to a passive layer, which is naturally formed on steel surface in the highly alkaline pore solution of concrete, have been precisely studied using versatile methods of measurements [13]. Laboratory tests and tests performed on facilities have demonstrated that elements of structures do not show any external symptoms of developing corrosion of reinforcing steel often for a very long time [14]. The literature basically describes three stages of corrosion degradation of reinforced concrete elements. At the first stage, corrosive agents gradually penetrate the porous structure of concrete cover and diffuse towards rebars. At the second stage, when corrosive agents reach the reinforcement surface, the passive layer is decomposed and corrosion processes are initiated on the steel surface. It is just the third stage when the volume of corrosion products (rust) is gradually increasing, corrosion damage to reinforced concrete in the form of cracks is visible, and finally spalling of smaller or bigger pieces of the cover is observed [15]. It is caused by tensile stresses formed in the concrete cover as a result of an expansion of corrosion products of steel and simultaneously very low tensile strength of concrete [16].

Today effects of degradation of reinforced concrete structures can be reduced or prevented using various methods. They include cathodic protection of reinforcement which provides permanent and controlled over time reduction of steel reinforcement potential in concrete to the level, at which metal exhibits resistance to corrosion. However, the use of the above method has to be preceded with the advanced diagnostic of corrosion employing such electrochemical methods as measurement of corrosion potential [17], measurement of the density of corrosion current [18]—the most common methods of linear polarization resistance (LPR) [19], electrochemical impedance spectroscopy (EIS) [20] and galvanostatic pulse (GP) method [21]. Those diagnostic methods are necessary for switching on and maintaining the direct current for cathodic protection throughout the whole service life of the structure. Another solution for increasing the durability of reinforcement in concrete elements of structures is the application of rebars made of stainless steel [22]. This type of steel, apart from basic components, such as iron and carbon, always contains at least 12% of chromium. Reinforcement for concrete structures is usually manufactured from austenitic, ferritic, or duplex (austenitic ferritic) steel [23]. Reinforcing steel can be replaced with non-metallic reinforcement made of FRP (Fiber-Reinforced Polymer) composite rebars. This type of reinforcement eliminates the problem of electrochemical corrosion [24,25]. Currently, three variants of those rebars are used in the construction sector: CFRP—with carbon fibers, GFRP—with glass fibers [26], and AFRP—with aramid fibers. However, non-metallic reinforcement in the construction sector has so far been rarely used when compared to traditional steel reinforcement.

In fact, protective epoxy and zinc coating is the latest possibility of direct protection of reinforcing steel in concrete against corrosion. Epoxy coatings on steel reinforcement in concrete behave like a typical electric insulator, which as a plastic material acts as a hardly permeable barrier cutting off steel from corrosive agents present in the concrete cover [27]. Unfortunately, technological processes occurring when reinforcement is laid in the formwork, and the process of concreting can cause point defects in those coatings. Places, where defects were produced, often invisible during construction works, can form local centers of corrosion [28,29] with time and concrete exposure to an aggressive environment. Considering the theory of reinforced concrete and structural mechanics, locally weakened reinforcing steel will also affect the local drop in load-carrying capacity of a reinforced concrete element, and consequently will pose a risk to the structure safety.

Zinc coatings prepared in HDG (Hot Dip Galvanizing) process [30,31,32,33] are free from drawbacks of epoxy coatings. In that case, any damage caused during the transport of reinforcement or its placing in the formwork does not create any risk for anti-corrosion protection of rebars in concrete [34]. The reason for no adverse effects is the formation of Fe-Zn alloy coating during the HDG process, which creates a galvanic cell in the concrete pore solution. As zinc is less noble regarding electrochemical series of metals, reinforcing steel is still protected against corrosion even in case of its punching [35]. It is a so-called “second phase of protection”, in which the zinc coating takes the role of an electrochemically dissolving electrode, and iron serves as the cathode. And impenetrable zinc coating insulates rebar from humidity (water) and oxygen during the first phase of protection. Studies indicate the thickness of zinc coating properly prepared in the HDG process, usually ranges between 50–300 µm [36]. However, the recommended thickness should not be smaller than 80 µm. The disadvantage of zinc coating is its possible corrosion after the contact with highly alkaline pore solution at pH < 11.4 and pH ≥ 13.3 [37,38]. This unfavorable occurrence is usually observed in the initial phase of bonding fresh concrete mix in the formwork, in which galvanized reinforcement is laid [39]. In this context it is crucial that there is only one-side limit of pH values for black reinforcing steel—pH < 11.8, below which corrosion develops. The effect of concrete carbonation on galvanized steel depends on the neutralisation degree of pore solution, in which CO_2_ gas dissolves [40]. If carbon dioxide acidifying the pore solution reduces pH values below 11.4, then zinc dissolution is initiated. However, according to Reference [37], zinc corrosion is unimportant, just minimal in this situation. As in case of black steel, the most destructive corrosion is observed on the coating of chloride ions. For the zinc coating, the limit content of chlorides is greater [37,41] than the one determined for black steel—0.4% [42] with reference to cement weight in concrete. For example, the boundary level equal to 1.5% is found in Reference [43], whereas Reference [44] mentions the percentage range of 1%–2%. Other studies did not define the percentage values of limit concentration, but specified chloride content initiating corrosion of zinc coating on the reinforcement as 2.5× [45] or, according to Reference [46] 4×–5× greater than the limit concentration determined for black steel. Discussions about limit values of chloride concentration unfavorable for the zinc coating manifest the difficulty in specifying any representative value. Such a great spread of values depends on the various compositions of concrete, mortar and grout used in tests. Moreover, different types of additives in concrete, different thickness of zinc coating and variation in chemical and phase composition of the coating across its thickness also affect corrosion damage.

The majority of described studies on galvanized reinforcing steel embedded in concrete refers to short time periods, that is, a few or more than ten weeks. The authors of this paper decided to perform comparative tests on the effectiveness of protection against chloride corrosion of zinc-coated low-carbon reinforcing steel of grade B500SP in concrete within over ten months. Apart from fundamental electrochemical polarization tests employing LPR and EIS techniques, the concrete-coating-reinforcement contact was additionally evaluated after two years of measurements using scanning electron microscopy (SEM) and energy dispersive spectroscopy (EDS) techniques.

## 2. Materials

Twelve concrete test elements with dimensions of 40 mm × 40 mm × 140 mm, reinforced with single steel bars were prepared for tests. Two batches of standard concrete mixes were used to place the concrete [47]. In both cases the mix contained 489 kg/m^3^ of CEM I 42.5—SR3/NA cement, 1669 kg/m^3^ of sand and aggregate of grain size of 2–8 mm. Water-cement ratio (*w*/*c*) was 0.45. About 3% of CaCl_2_ by cement weight, dissolved in batched water, was added to one of those mixes. In conclusion, six test elements were prepared from concrete without any additives, and another group of six specimens was prepared from concrete with chloride additives.

Each concrete test element was reinforced with a single ribbed rebar with a diameter of ϕ8 mm, made of steel of grade B500SP classified to steel class C of high ductility [48]. As claimed by the manufacturer, maximum values of additives to low-carbon steel for elements, in which content exceeds 0.5% should not be greater than 1.65% for Mn, 0.85% for Cu, and 0.6% for Si.

In accordance with the primary purpose of studies, half of rebars made of steel of grade B500SP, used as the reinforcement in concrete test elements, was coated in the HDG process. For that purpose, holders were welded to cleaned bars cut to 1.5 m long pieces to provide galvanizing in a vertical position. Rebars placed on plating racks were successively immersed in a degreasing, pickling and finally fluxing bath. Prior to the immersion in the galvanizing bath, rebars were dried in a dryer. Bathing in molten zinc, at a temperature of ca. 450 °C, lasted 120 s. The obtained zinc coatings on steel rebars had an average thickness of ca. 100 µm, which met standard requirements [49,50]. Before the tests, the thickness of the zinc coating was initially verified under the metallographic microscope Delta Optical MET-200-TRF (Delta Optical Ltd., Nowe Osiny, Poland).

Before concreting the specimens, galvanized or black steel rebars were cut into sections of 15 cm in length. Ends of each rebar were protected with shrink film against crevice corrosion in zones, where rebars expanded from concrete. Then, the rebars were stabilized in standard moulds for preparing specimens of masonry mortar, and then concreted. After two days from concreting, the specimens were stripped from the mould. Two weeks after concreting, polarization measurements of the reinforcement began.

**Figure 1:**
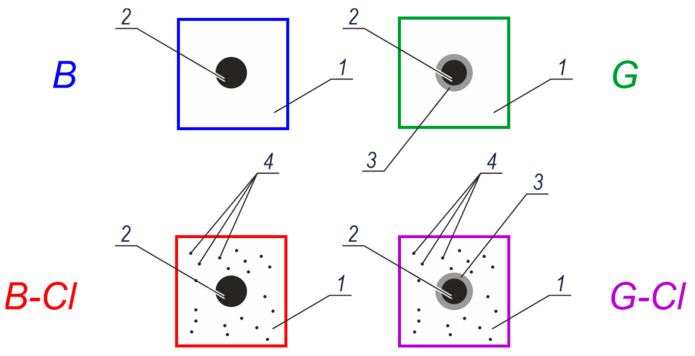
Four groups of reinforced concrete test elements used in tests: 1—concrete without additives, 2—black steel, 3—zinc coating on reinforcing steel, and 4—chlorides in concrete.

In summary, six concrete test elements were reinforced with galvanized rebars, and other six with black steel rebars. As two concrete mixes were used during tests, the total number of twelve test elements was divided into four groups, each of them containing three identical specimens. To differentiate clearly those groups, different colors and symbols were assigned to each of them as shown schematically in Figure 1:

- blue color and *B* symbol—black steel in concrete without any additives,
- green color and *G* symbol—galvanized steel in concrete without any additives,
- red color and *B-Cl* symbol—black steel in concrete with chlorides,
- violet color and *G-Cl* symbol—galvanized steel in concrete with chlorides.

Assigned symbols and colors are consequently used in this paper to describe and analyze performed tests.

## 3. Test Methods

### 3.1. Electrochemical Polarization Tests

**Figure 2:**
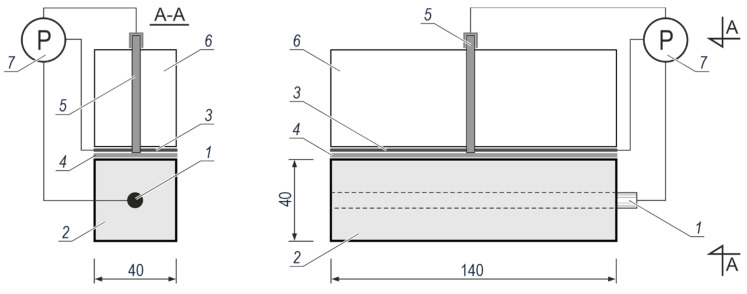
Measuring system for polarization tests of steel reinforcement in concrete: 1—working electrode, 2—concrete, 3—auxiliary electrode, 4—wet felt, 5—reference electrode Cl^−^/AgCl,Ag, 6—ballast with a guide for reference electrode, 7—potentiostat.

Polarization measurements of the reinforcement in concrete test elements were conducted in a three-electrode arrangement illustrated in Figure 2. Galvanized or black steel rebar 1 embedded in concrete 2 with or without chloride additives served as the working electrode. The auxiliary electrode 3 was made of a 2 mm thick stainless-steel sheet having the dimensions of 140 mm × 40 mm. A wet felt 4 with the dimensions of 140 cm × 40 cm provided the electric contact between the sheet 3 and the top surface of concrete. The reference electrode 5 (Cl^−^/AgCl,Ag) was placed in a vertical ballast guide 6 to provide its stabilisation during tests. The ballast 6 was used to provide the proper adhesion of the auxiliary electrode 3 through the felt 4 to the top surface of the concrete specimen 2. All three electrodes were connected to the potentiostat 7 Gamry Reference 600.

Electrochemical tests were performed on the above measuring system using the following techniques: linear polarization resistance (LPR) and electrochemical impedance spectroscopy (EIS). The specimens were kept at ca. 20 ± 2 °C and the relative humidity of 50 ± 10%. Prior to beginning polarization tests on a given day, the top surface of the concrete specimen was immersed in tap water to the depth up to 5 ± 1 mm for ca. 30 min to ensure better conductivity of concrete cover in the tested reinforcement during measurements. After taking from water, the specimens were connected to the potentiostat *7* and changes of gradually stabilizing potential were observed with the reference electrode *5* for 60–120 min. When potential changes were at the level of 0.1 mV/s, EIS methods were performed on the steel reinforcement in concrete. Testing was performed at the fixed range of frequencies of 0.01 Hz–1 kHz in the potentiostatic mode with a disturbing sinusoidal signal at the potential amplitude of 10 mV over the corrosion potential. Then, after a few minutes or a longer interval when changes in the corrosion potential did not exceed the level of 0.1 mV/s, LPR tests were conducted in the potentiodynamic mode. The reinforcement was polarized at a rate of 1 mV/s within the range of potential changes from −150 mV to +50 mV regarding the corrosion potential.

The first measuring series began two weeks after placing specimens in concrete. Other five measuring series were conducted in one- and two-week intervals. At the next stage, six measuring series were taken at a two-week interval. Final measuring series took place in a month interval. After 18 months, prior to scheduled microscopic measurements, EIS and LPR tests were repeated but only on four specimens representing each of *B*, *G*, *B-Cl*, and *G-Cl* groups. At the final testing stage, results obtained from polarization measurements were compared with microscopic observations and analyses.

### 3.2. Structural Tests

The specimens for structural tests were cut out perpendicularly to the rebar axis. Metallographic specimens were prepared from those cut pieces of concrete with rebars made of steel B500SP. Tests on the macrostructure of concrete specimens were performed at the stand specially prepared for macro testing. And microstructure of concrete was tested using Olympus SZX9 stereo microscope and GX51 inverted microscope (LM) (Olympus Corporation, Tokyo, Japan).

Advanced tests on the specimen structure at selected points were performed with HITAHI S-3400N scanning electron microscope (SEM) (Hitachi, Tokyo, Japan) at an accelerating voltage within the range of 15–25 kV. Tests on chemical composition at selected micro-areas of the specimens were performed with the energy dispersive spectroscopy (EDS) using the spectrometer by THERMO NORAN company with SYSTEM SIX software (Thermo Fisher Scientific Inc., Waltham, MA, USA). As microscopic tests are invading into the structure of the specimen material, they were performed after the final stage of electrochemical tests. Therefore, those tests should be only related to results obtained after the last, 792 day of measurements.

## 4. Results from Electrochemical Tests and Their Analysis

### 4.1. Measuring Corrosion Potential of Reinforcement in Concrete

**Figure 3:**
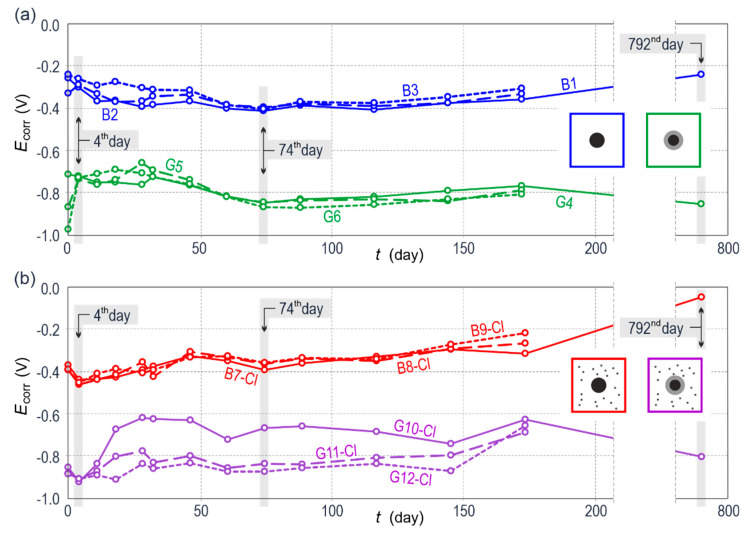
Changes in the function of time for corrosion potential *E*_corr_ of galvanized and black steel reinforcement (**a**) in concrete without chlorides, (**b**) in concrete with chlorides; selected measuring series presented as diagrams and analyzed in details as part of electrochemical impedance spectroscopy (EIS) and linear polarization resistance (LPR) measurements are marked with grey color.

Figure 3 shows a comparison of results from 160 measurements of corrosion potential of the steel reinforcement in concrete from twelve test elements. Figure 3a presents results for concrete without any additives, whereas Figure 3b refers to concrete with calcium chloride. All measurements were performed against the silver chloride electrode.

Core tests were conducted in less than six months and restarted after about an 18-month interval, but only on four selected specimens that were further subjected to microscopic tests.

### 4.2. Linear Polarization Resistance (LPR) Tests

**Figure 4:**
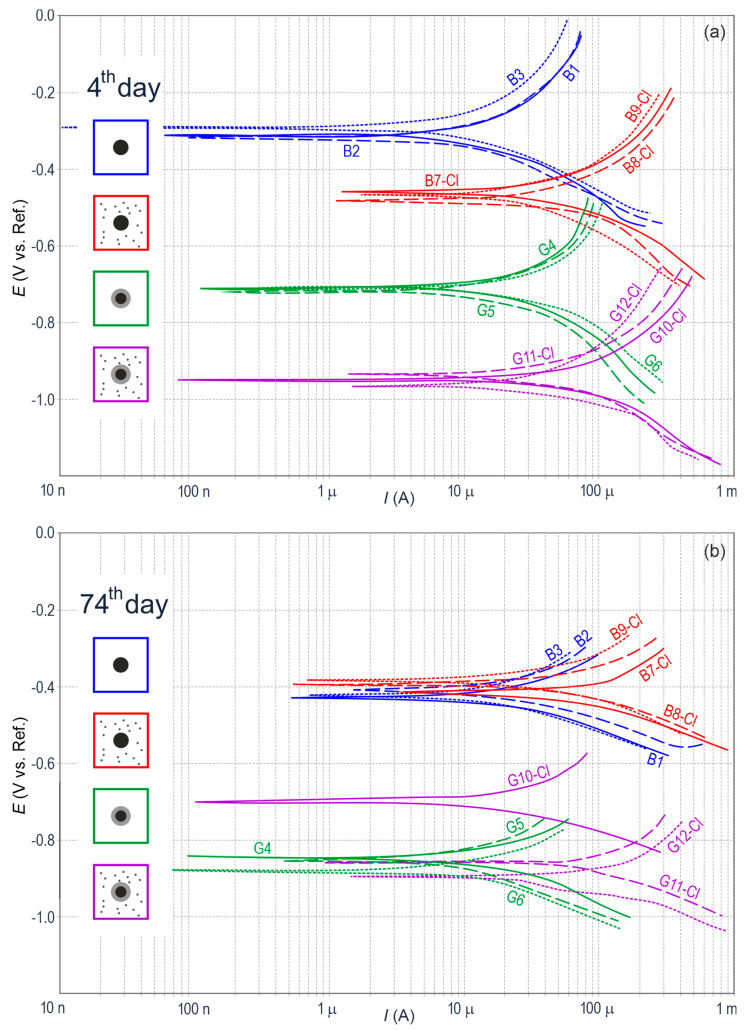
Potentiodynamic polarization curves for steel reinforcement in concrete from 12 specimens (**a**) on day 4 and (**b**) on day 74 of measurements: *B*—black steel in concrete without additives, *G*—galvanized steel in concrete without additives, *B-Cl*—black steel in concrete with chlorides, and *G-Cl*—galvanized steel in concrete with chlorides.

Linear polarization resistance (LPR) tests were the last tests from the group of electrochemical tests performed on the reinforced concrete specimen. Contrary to electrochemical impedance spectroscopy (EIS) tests regarded as complementary measurements, LPR tests were conducted on each test element from each measuring series. The whole period of testing produced a total of 160 polarization curves, in which exemplary shapes for three selected measuring series are illustrated in Figure 4. Characteristic time points for measurements were the following measuring days: 4, 74, and 792. The selected measuring days, marked with grey color in Figure 3, were to provide a more detailed presentation and analysis of test results from the initial phase of measurements (day 4), the halfway point of tests (day 74) and from the period directly before planned microscopic tests and analyses (day 792).

On day 4 of measurements, shapes of polarization curves (Figure 4a) were more arranged showing the clear formation of four groups. The corrosion potential of the specimens with black steel was less negative than in case of galvanized steel. The mean potential value in the specimen groups *B* and *B-Cl* was −0.28 V and −0.45 V, respectively. For the groups *G* and *G-Cl*, it was equal to −0.73 V and −0.91 V, respectively. However, the most significant information obtained from the analysis of polarization curves was the polarization resistance *R*_p_ was inversely proportional to the density of corrosion current *i*_corr_. The Stern-Geary Equation (1) was used to calculate values of *i*_corr_, taking into account coefficients of rectilinear slope for segments of polarization curves—anodic *b*_a_ and cathodic *b*_c_.

(1) $$i_{\mathrm{corr}}=\frac{B}{R_{p}\cdot A},\;B=\frac{b_{a}b_{c}}{2.303\cdot \left(b_{a}+b_{c}\right)}$$

Parameters of the Equation (1) taken for calculations of *i*_corr_ are shown in Appendix A—Table A1, which contains results for only 28 out of 160 analyzed polarization curves. Mean densities of corrosion current for individual groups used further in the paper, demonstrated that the lowest value of *i*_corr,mid_ = 0.31 µA/cm^2^ was obtained for the specimens from the group *B*. Because of naturally high alkaline pH (pH > 12.5) of concrete pore solution without additives, the obtained current density could indicate the passivation of reinforcing steel in concrete. Adding calcium chloride to the concrete mix caused the same black steel started to corrode in the specimens from the group *B-Cl* with *i*_corr.mid_ = 1.65 µA/cm^2^. And the zinc coating on the reinforcement also began to dissolve electrochemically at pH > 13.3 when in direct contact with highly alkaline pore solution. The mean density of corrosion current *i*_corr.mid_ = 0.93 µA/cm^2^ calculated for the group *G* could with high probability indicate that occurrence typical for the initial phase of concrete curing. The final, the highest mean density of corrosion current *i*_corr.mid_ = 1.98 µA/cm^2^ referred to galvanized reinforcing steel in concrete with chloride additives. For the specimen group *G-Cl*, it could indicate the accumulation of zinc corrosion caused by high pH of concrete pore solution and aggressive impact of chloride ions.

On day 74 of measurements, distributions of polarization curves (Figure 4b) were slightly shifted when compared to the distribution of the same specimens presented in Figure 4a. The corrosion potential of black steel in concrete without chlorides, averaged for the same group of specimens, was considerably reduced to *E*_corr.mid_ = −0.40 V, and in concrete with chlorides the potential slightly increased to *E*_corr.mid_ = −0.37 V. For galvanized steel in concrete without chlorides, a clear drop in potential to the value *E*_corr.mid_ = −0.85 V was observed with the simultaneous increase in potential to the following level *E*_corr.mid_ = −0.79 V. The comparison of graphical distributions of polarization curves from the beginning of tests (Figure 4a) and the halfway point of measurements (Figure 4b) can suggest that corrosion potential of black reinforcing steel had similar values as in case of galvanized steel, but with values at a greater spread.

The analysis of calculated mean densities of corrosion current on day 74 of measurements indicated in concrete with chlorides still low values of *i*_corr.mid_ = 0.27 µA/cm^2^ for black steel and a dramatic drop in *i*_corr.mid_ to 0.14 µA/cm^2^ for galvanized steel. The passivation of black steel in concrete without additives was a natural process, but no corrosion of zinc coating on steel reinforcement after several dozens of days in contact with the pore solution was desired despite the initially intensive dissolution of zinc. In concrete specimens with chlorides, the corrosion of black steel *i*_corr.mid_ = 0.71 µA/cm^2^ and galvanized steel *i*_corr.mid_ = 1.16 µA/cm^2^ was still observed on day 74 of measurements. But mean densities of corrosion current were slightly lower than on the first days of measurements.

**Figure 5:**
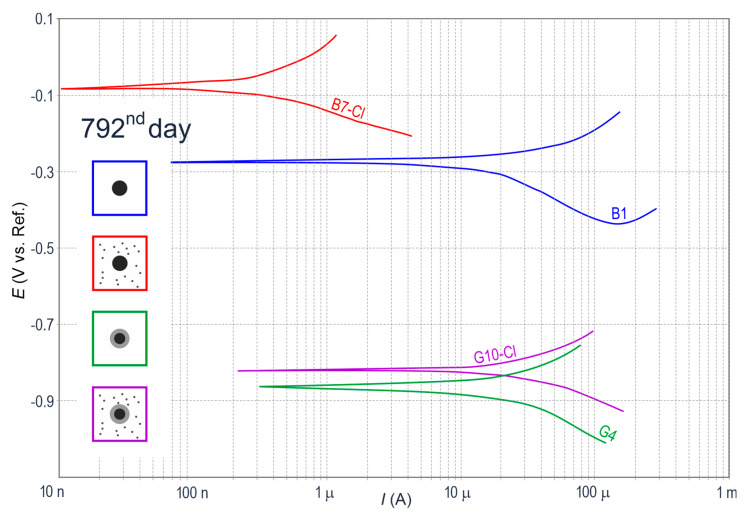
Potentiodynamic polarization curves for the steel reinforcement in concrete from four specimens on day 792, directly before microscopic tests: *B1*—black steel in concrete without additives, *G4*—galvanized steel in concrete without additives, *B7-Cl*—black steel in concrete with chlorides, and *G10-Cl*—galvanized steel in concrete with chlorides.

After about two years of measurements (day 792), prior to the commencement of microscopic tests, polarization curves for individual specimens representing each of four groups (Figure 5) showed an interesting change. A noticeable change was found for black steel in concrete with chlorides (*B7-Cl*) because its corrosion potential largely increased to −0.04 V, but what is more important, the density of corrosion current dramatically decreased to just 0.01 µA/cm^2^. Taking into account the fact the analyzed reinforced concrete specimen was potentially the most vulnerable to corrosion, the only possible explanation for the above situation was the high likelihood of the large accumulation of corrosion products (rust) around the steel rebar, and consequently, a serious limitation of direct access for oxygen and humidity to its surface. Hence, electrode processes on steel were stopped. For galvanized reinforcing steel in concrete with chlorides *(G10-Cl),* the corrosion current density dropped to 0.34 µA/cm^2^. The likely reason was the accumulation of corrosion products of zinc on the coating slightly slowing down its further dissolution. After two years of measurements, black steel (*B1*) and galvanized steel (*G4*) in the concrete specimens without chlorides had stable but low density of corrosion current (0.35 µA/cm^2^ and 0.29 µA/cm^2^, respectively), which suggested the reinforcement corrosion was not likely to develop.

### 4.3. Electrochemical Impedance Spectroscopy (EIS) Tests

Electrochemical impedance spectroscopy (EIS) tests on the specimens of concrete with reinforcement were additional to core polarization tests performed by linear polarization resistance (LPR) method. Impedance tests focused only on three selected measuring series on day 4, 74, and 792.

**Figure 6:**
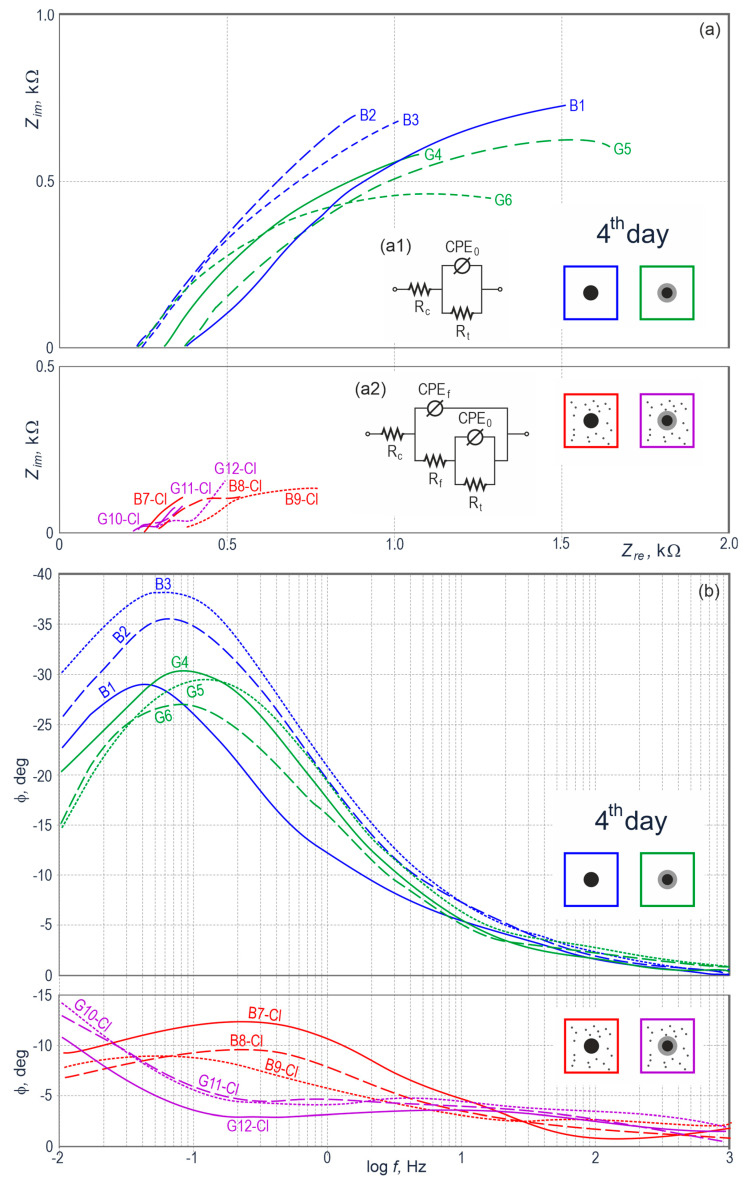
Results from impedance testing of steel reinforcement in concrete from 12 specimens on day 4 of measurements illustrated at the Nyquist (**a**) and Bode (**b**) plots, *B*—black steel in concrete without additives, *G*—galvanized steel in concrete without additives, *B-Cl*—black steel in concrete with chlorides, *G-Cl*—galvanized steel in concrete with chlorides; (**a1**) electrical equivalent circuit for the *B* and *G* spectrum groups, (**a2**) electrical equivalent circuit for the *B-Cl* and *G-Cl* spectrum groups.

Figure 6a illustrates in the complex plane results from impedance tests on the steel reinforcement in concrete from 12 specimens on day 4 of measurements, whereas Figure 6b presents the same results as the function of phase shift angle *ϕ* against the logarithm of measurement frequency *f*. For the specimens from groups *B* and *G* made of concrete without chlorides, spectra at the Nyquist plots (Figure 6a) exhibited capacitive behavior similar in shape to parts of semi-circles, in which extrapolation to the horizontal axis of real impedance provided the possibility of estimating charge transfer resistance *R*_t_ across the interface. It is interesting to note that semi-circles referring to galvanized steel had slightly smaller conventional diameter. The probable reason was the dissolution of the zinc coating in highly alkaline (pH > 13.3) of concrete pore solution.

For the specimens from groups *B-Cl* and *G-Cl* made of concrete with chlorides, the size of spectra at the Nyquist plot was much smaller and the second time-constant was clearly noticeable. Shapes of those spectra were rather characteristic for the development of metal (Fe or Zn) corrosion in concrete. Observations made for the Nyquist plot (Figure 6a) were additionally confirmed by the Bode plot (Figure 6b). Low frequencies typical for metal in concrete without additives (groups *B* and *G*) displayed quite wide angles of phase shift (−20°–−40°), whereas the specimens of concrete with chlorides (groups *B-Cl* and *G-Cl)* reached the maximum level of −15°. The observed shapes of those spectra, which were very distinctive, quite explicitly indicated the corrosion of metal in the specimens from groups *B-Cl* and *G-Cl*. For galvanized steel (group *G*) the situation was slightly less favorable, which could suggest not so intensive processes of zinc dissolution.

The above qualitative analysis of obtained spectra was confirmed by the quantitative analysis. For that purpose, the equivalent electrical circuit illustrated in Figure 6(a1), was proposed for spectra of concrete specimens without chlorides. It was the classic Randles circuit, in which the double layer capacity was replaced with a constant phase element *CPE*_0_. Parameter *R*_c_ is resistance of concrete, *R*_t_ is charge transfer resistance across the interface. The electric circuit shown in Figure 6(a2), which simulated the porous coating, was used for concrete specimens with chlorides. Besides already mentioned parameters of the Randles circuit, that circuit had two characteristics of the electrochemical zinc coating on the steel reinforcement: *R*_f_—resistance of the coating layer and *CPE*_f_—constant phase element characterizing its quasi capacity. The Simplex method was used to find numerical values of elements in equivalent circuits, and those values are presented in Appendix A—Table A2. The summary table presenting calculated results, also contain numerical parameters of constant phase elements: *CPE*_0_—*Y*_0_ and α_0_ and *CPE*_f_—*Y*_f_ and α_0_.

**Figure 7:**
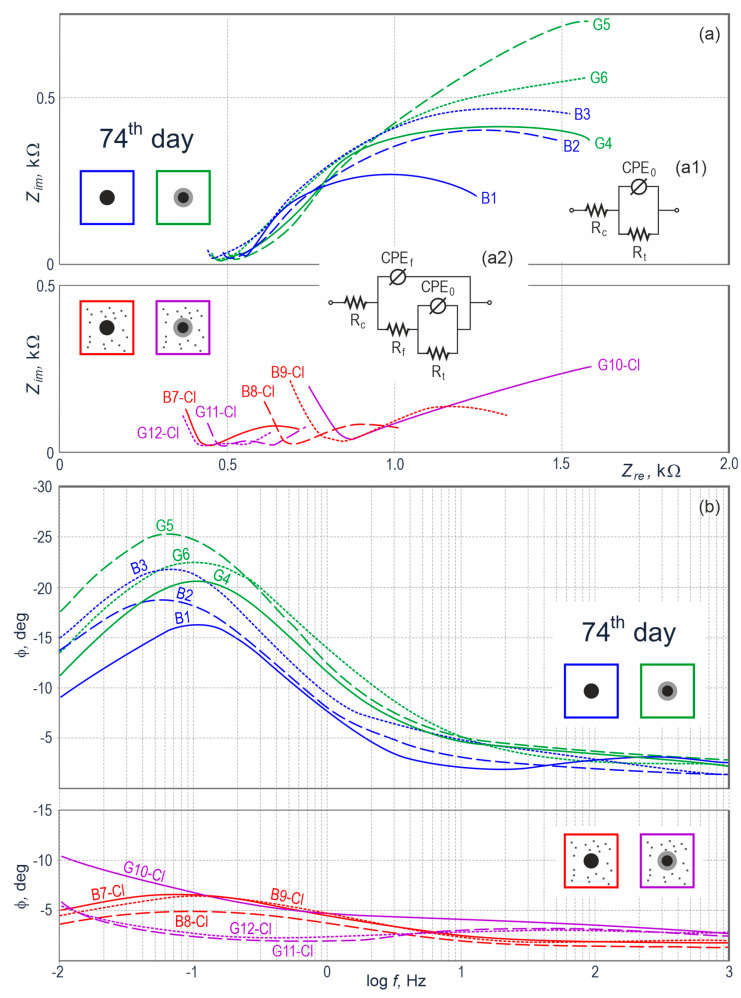
Results from impedance testing of steel reinforcement in concrete from 12 specimens on day 74 of measurements illustrated at the Nyquist (**a**) and Bode (**b**) plots, *B*—black steel in concrete without additives, *G*—galvanized steel in concrete without additives, *B-Cl*—black steel in concrete with chlorides, and *G-Cl*—galvanized steel in concrete with chlorides; (**a1**) electrical equivalent circuit for the *B* and *G* spectrum groups, (**a2**) electrical equivalent circuit for the *B-Cl* and *G-Cl* spectrum groups.

On day 74 of measurements, the impedance spectra at the Nyquist plot (Figure 7a) obtained for all four groups of the specimens were similar to spectra from day 4 (Figure 6a). The main difference referred to the specimens with galvanized steel, for which the spectra at the Nyquist plot had a slightly different shape resembling a typical image of metal passivated in concrete at low frequencies. That observation was confirmed by the Bode plot (Figure 7b), at which phase shift angles of galvanized steel at low frequencies were greater by ca. 5° (with reference to absolute values) than for black steel. Relationships between shapes of impedance spectra were rather typical for zinc behavior in hardened concrete without any additives.

For the specimens of concrete with chlorides, day 74 of measurements revealed a small drop in the intensity of corrosion processes of galvanized and black steel. The presented deduction resulted from the quantitative analysis on the basis of the equivalent electrical circuits illustrated in Figure 7(a1,a2), similar to diagrams shown in Figure 6(a1,a2). That phenomenon could be explained by a gradual accumulation of corrosion products of iron and zinc on the surface of steel reinforcement in the specimen, which additionally were deposited in concrete pores. Dissolution of metals (Fe and Zn) was slightly inhibited mainly due to the limited access of oxygen to the surface of both metals.

**Figure 8:**
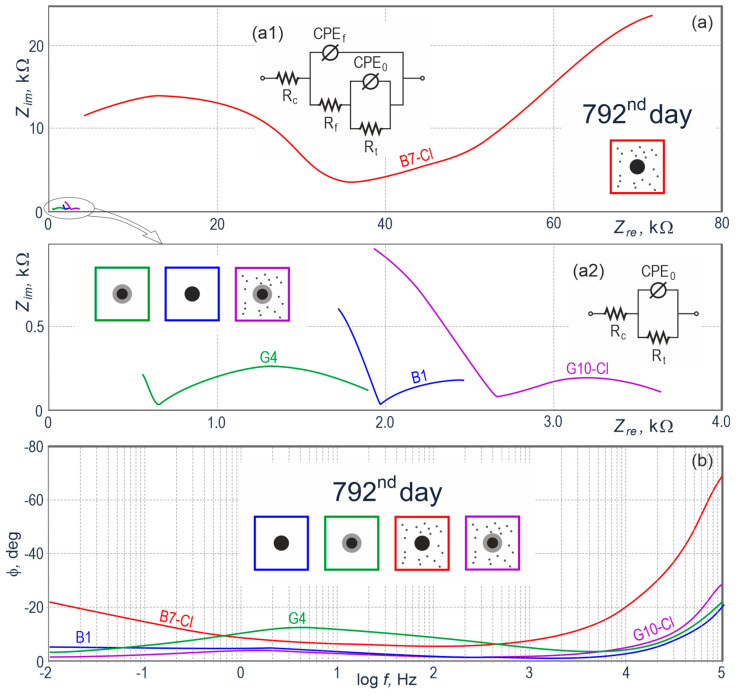
Results from impedance testing of steel reinforcement in concrete from 12 specimens on day 792 of measurements illustrated at the Nyquist (**a**) and Bode (**b**) plots, directly prior to microscopic tests *B1*—black steel in concrete without additives, *G4*—galvanized steel in concrete without additives, *B7-Cl*—black steel in concrete with chlorides, and *G10-Cl*—galvanized steel in concrete with chlorides; (**a1**) electrical equivalent circuit for the *B-Cl* spectra group, (**a2**) electrical equivalent circuit for the *B, G* and *G-Cl* spectrum groups.

The last series of impedance measurement was made on day 792, that is, after about two years of measurements and included only four specimens (*B1*, *G4*, *B7-Cl*, *G10-Cl*) being the one-element representation of all four groups (*B*, *G*, *B-Cl*, *G-Cl*) of test elements. The Nyquist plot (Figure 8a) shows one dominating spectrum (*B7-Cl*) and three considerably smaller spectra near the beginning of the coordinate system. For better clearness of the results, those three spectra are again presented below, in the second complex plane with 20-time smaller values of complex and real impedance. The clear dominance of one spectrum over others indicated the impedance of the whole steel-concrete system in the specimen *B7-Cl* was about 20 times greater than the impedance of the other three specimens. As all impedance measurements on the final measuring series (day 792) were conducted within a broader range of frequencies 0.01 Hz–100 kHz, particularly the spectrum *B7-Cl* had a rather interesting shape. It had (from the left side) a high-frequency, slightly oblate semi-circle expressing electrochemical properties of concrete, and from the point of contraflexure a rectilinear part changing into the arc shape. The last described part could indicate diffusion control over the corrosion process. The spectrum shape for *B7-Cl* in the complex plane and at the Bode plot demonstrated a very strong slowdown on day 792 of measurements, comparable to the situation, in which dissolution of reinforcing steel in concrete was inhibited despite the presence of aggressive chloride ions. A continuous increase in the volume of corrosion products was supposed to inhibit seriously the access of oxygen to the steel surface, and consequently to eliminate the agent required for the development of electrochemical corrosion of steel in concrete.

The qualitative evaluation and adjustment with the Simplex method of the theoretical spectrum, according to the diagram shown in Figure 8(a1) confirmed the above observations as the determined charge transfer resistance *R*_t_ reached 1680 kΩcm^2^, which corresponded to the very low density of corrosion current *i*_corr_ = 0.01 µA/cm^2^

Impedance spectra of the other three specimens (*B1, G4, G10-Cl*) at the Nyquist plot were rather similar in shape, particularly with reference to the low-frequency range describing the properties of metal in concrete. The qualitative analysis conducted with the electrical equivalent circuit illustrated in Figure 8(a2) also confirmed similar values of charge transfer resistance *R*_t_ = 34 k–50 kΩcm^2^, which corresponded to very low densities of corrosion current *i*_corr_ = 0.29–0.35 µA/cm^2^. According to criteria published in [51], determined for rebars in concrete, such low densities of corrosion current usually indicate a very low level of steel corrosion. Moreover, each spectrum had a noticeably different inflexion point (that is, the closest point to the horizontal axis of real impedance), from which electrochemical properties of concrete were revealed within the low-frequency range. Diversified inflection points of those spectra indicated different values of concrete resistivity during measurements under the impact of alternating current flowing between reinforcing steel and the auxiliary electrode, placed on the top surface of the concrete specimen. The analyzed cases showed concrete in the specimen *G4* had the lowest resistivity, and concrete in the specimen *G10-Cl* had the highest resistivity.

## 5. Results from Structural Tests and Their Analysis

Corrosion tests indicated the change in parameters of corrosion current over time which could suggest structural changes at the concrete-rebar interface. Therefore, microstructure and chemical compositions at the interface between concrete and rebar made of steel B500SP were analyzed for all four tested cases after completing corrosion tests:

- black reinforcing steel B500SP in concrete without any additives,
- galvanized reinforcing steel B500SP in concrete without chlorides,
- black reinforcing steel B500SP in concrete with chlorides
- galvanized reinforcing steel B500SP in concrete with chlorides.

### 5.1. General Description of the Microstructure of Concrete Reinforced with Rebars Made of Steel B500SP (Stereo, LM)

**Figure 9:**
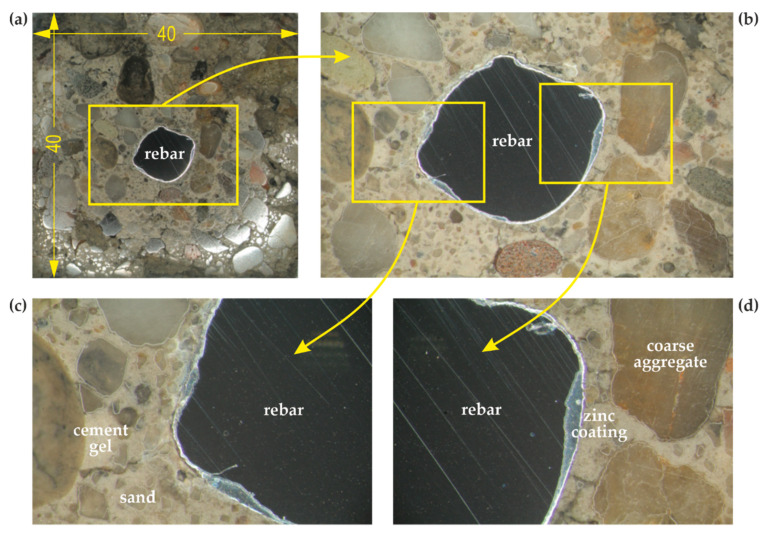
Cross-section of the concrete specimen with dimensions of 40 mm × 40 mm, containing galvanized rebar (stereoscope): (**a**) general view of a cross-section of a steel bar in concrete (**b**) detail marked with a rectangle from Figure 9a, (**c**,**d**) detail marked with a rectangle from Figure 9b.

Tests on concrete-reinforcement joints began from analyzing its microstructure after completing corrosion tests, using the stereo microscope (Figure 9). Figure 9a presents the cross-section (with dimensions of 40 mm × 40 mm) of the tested concrete specimen with a galvanized rebar concreted in the central point, made of ribbed steel of grade B500SP and with a diameter of ϕ8 mm.

Figure 9b illustrates the close-up of the cross-section of steel rebar with a clearly visible, thin, silver and circumferential zinc coating. There are noticeable particles of coarse and fine (sand) aggregates near the rebar. Aggregates are tied together with cement gel. Figure 9c,d show another close-up of a strongly diversified thickness of the zinc coating, whose thickest layer is observed near oblique ribs of the reinforcing steel.

**Figure 10:**
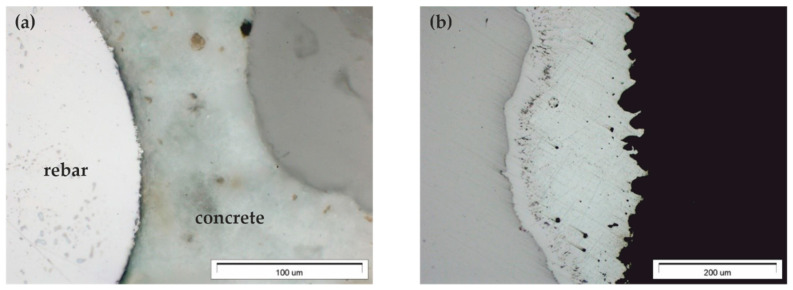
Microstructure of the joint between concrete and rebar made of galvanized steel B500SP (LM): (**a**) general view of a cross-section of a steel bar in concrete (**b**) morphology of the zinc coating on the rebar in concrete.

The tests conducted on the microstructure of reinforced concrete specimens using the light microscope revealed good conditions of joints between concrete and the reinforcement for rebars made of black steel (Figure 10a). And simultaneous observations confirmed previous conclusions made on the basis of tests using the stereo microscope (Figure 9) concerning the non-uniform thickness of zinc coating on rebars (Figure 10b).

### 5.2. Evaluation of Microstructure and Chemical Compositions of Joints Between Concrete and Rebar (SEM, EDS)

#### 5.2.1. Black Reinforcing Steel B500SP in Concrete with and without Chlorides

**Figure 11:**
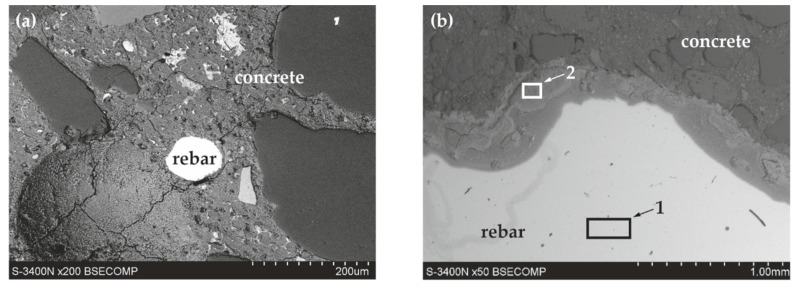
Microstructure of the joint between concrete without chlorides and reinforcement made of black steel B500SP (SEM): (**a**) general view of a cross-section of a steel bar in concrete (**b**) morphology of the boundary of separation rebar–concrete with marked places 1 and 2 for microanalysis of the chemical composition.

**Figure 12:**
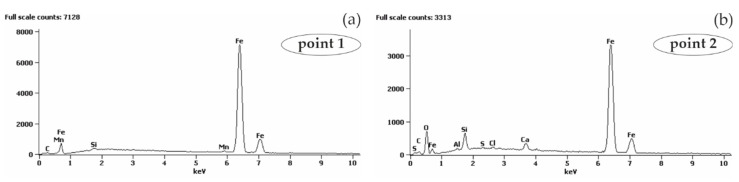
Spectra obtained from energy-dispersive X-ray spectroscopy (EDS) method for areas marked in Figure 11b: (**a**) from point 1 (**b**) from point 2.

The analysis of the microstructure of joints between concrete without chlorides and rebar made of B500SP black steel (Figure 11) showed a difference in concrete structure regarding its phase composition (Figure 11a—image from BSE detector) and rebar correctly embedded in concrete. The analysis of chemical composition at the interface between concrete and rebar (Figure 12) indicated the presence of elements being components of concrete or steel rebar. Oxygen (Figure 12b), probably in the form of oxide, was found on the rebar surface. Oxidation of zinc coating is a characteristic effect of its destruction [52].

As expected, no traces of corrosion damage at the interface between steel and concrete were found, what was normal in the highly alkaline environment of concrete. Results from polarization tests on corrosion current density also indirectly suggested the passivation of steel, which was typical for such a system.

**Figure 13:**
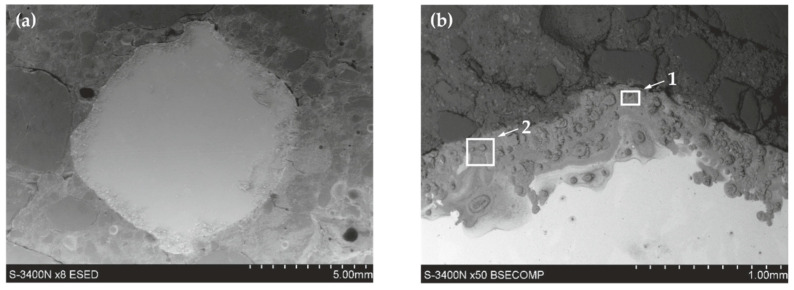
Microstructure of the joint between concrete with chlorides and black reinforcing steel B500SP (SEM): (**a**) general view of a cross-section of a steel bar in concrete (**b**) morphology of the boundary of separation rebar – concrete with marked places 1 and 2 for microanalysis of the chemical composition.

**Figure 14:**
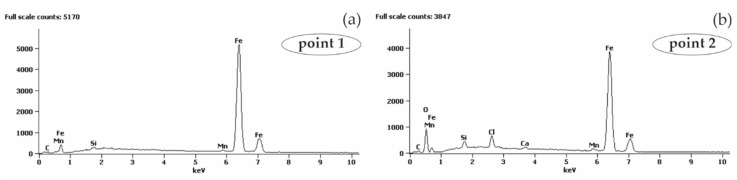
Spectra obtained from energy-dispersive X-ray spectroscopy (EDS) method for areas marked in Figure 13b: (**a**) from point 1 (**b**) from point 2.

The analysis of joints between concrete with chlorides and black reinforcing steel B500SP indicated a clear change in the nature of the joint when compared to concrete without chlorides (Figure 11). The presence of chlorides in concrete resulted in a large number of corrosion products at the rebar/concrete interface (Figure 13). Corrosion products of the steel reinforcement (rust) did not only accumulate on the surface of the rebar, but also penetrated the porous structure of concrete. A considerable concentration of Cl and oxygen (Figure 14b) was found in these products which probably indicated the presence of chlorides and oxides in that area.

No concrete cracking in the interfacial zone was seen which indicated critical stresses blowing out the concrete cover from the inside did not occur. It should be noticed that the layer of corrosion products was thick enough to make the diffusion of oxygen to the steel surface very difficult. That effect was evident in results from impedance tests (Figure 8a) due to the appearance of the additional time constant.

#### 5.2.2. Reinforcing Steel B500SP with Zinc Coating, in Concrete with and without Chlorides

**Figure 15:**
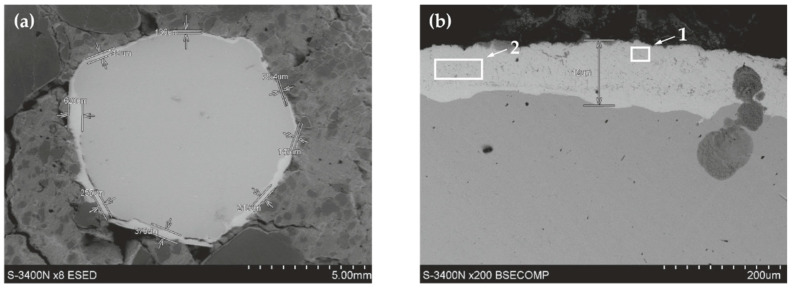
Microstructure of the joint between concrete without chlorides and reinforcing steel B500SP with zinc coating (SEM): (**a**) general view of a cross-section of a steel bar in concrete (**b**) morphology of the boundary of separation rebar - concrete with marked places 1 and 2 for microanalysis of the chemical composition.

**Figure 16:**
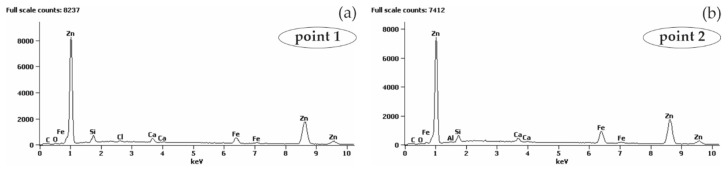
Spectra obtained from energy-dispersive X-ray spectroscopy (EDS) method for areas marked in Figure 15b: (**a**) from point 1 (**b**) from point 2.

**Figure 17:**
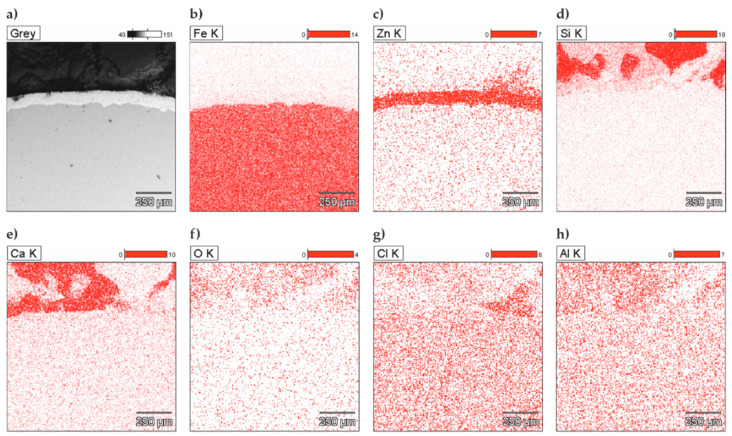
(**a**) Microstructure of the surface of tested concrete without chlorides—reinforcing steel B500SP with zinc coating (SEM) and relative concentrations of elements on the surface (mapping): (**b**) Fe, (**c**) Zn, (**d**) Si, (**e**) Ca, (**f**) O, (**g**) Cl, and (**h**) Al (EDS).

The analysis of the joint between concrete without chlorides and the reinforcing steel B500SP with zinc coating demonstrated a clear difference the coating thickness (Figure 15). It varied from more than ten (78.4 µm) to over 600 µm, but usually was within the range from 150 µm to 250 µm. The thickest zinc coating (620 µm) was near one of oblique ribs of the rebar. The variable thickness of the zinc coating is an effect directly resulting from the technology of the galvanizing process, but it can also be the result of the preparation of metallographic samples—the zinc “rubs” on the surface of the specimen. In this case, it is the effect of galvanizing technology. The analysis of chemical composition for the section of the zinc coating indicated its great uniformity (Figure 16). That result was confirmed by testing the relative concentration elements on the surface (mapping) of the concrete-rebar joint (Figure 17).

Polarization tests on the galvanized reinforcement in concrete without chlorides indicated some increase in corrosion current density on first days of measurements when compared to black steel. This effect is usually noticed during the first weeks of concrete curing. In this case, the microscopic analysis referred to a two-year old concrete, for which processes of dissolution of zinc coating on steel are not usually observed or have been stabilized. Therefore, it is difficult to determine the original thickness of the zinc coating. It may be wondered whether it’s very non-uniform thickness observed under the microscope was only caused by the specificity of the technological process of the formation of hot-dip galvanized zinc coating or the effect of partial dissolution of zinc in the highly alkaline environment of concrete pore solution.

**Figure 18:**
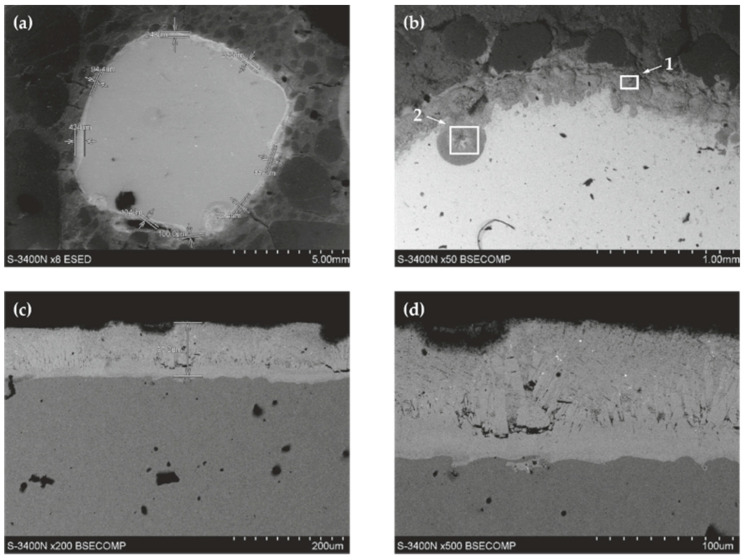
Microstructure of the joint between concrete without chlorides and galvanized reinforcing steel B500SP (SEM): (**a**) general view of a cross-section of a steel bar in concrete (**b**) morphology of the boundary of separation rebar–concrete with marked places 1 and 2 for microanalysis of the chemical composition (**c**) morphology of the zinc coating on the rebar in concrete (**d**) area of the zinc coating with the crack shown in Figure 18c.

The analysis of joint between concrete with chlorides and galvanized reinforcing steel B500SP indicated significant non-uniformity of the zinc coating on the rebar with reference to its thickness and morphology (Figure 18), and a similar situation was observed for the bond when no chlorides were present.

**Figure 19:**
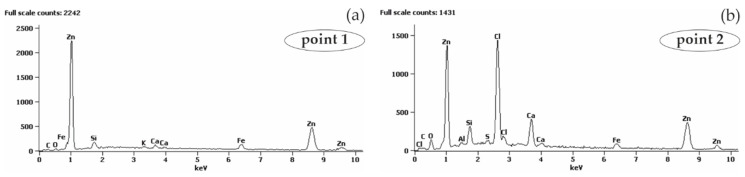
Spectra obtained from energy-dispersive X-ray spectroscopy (EDS) method for areas marked in Figure 18b: (**a**) from point 1 (**b**) from point 2.

**Figure 20:**
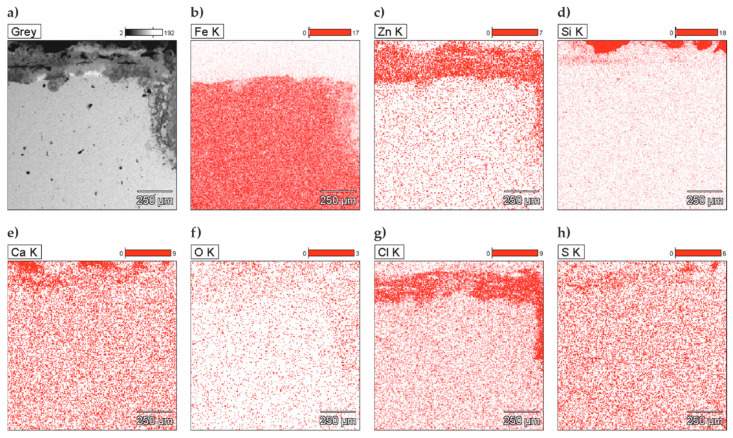
(a) Microstructure of the surface of tested concrete without chlorides—reinforcing steel B500SP with zinc coating (SEM) and relative concentrations of elements on the surface (mapping): (**b**) Fe, (**c**) Zn, (**d**) Si, (**e**) Ca, (**f**) O, (**g**) Cl, and (**h**) Al (EDS).

The thickness of the zinc coating varied from as low as 100 µm (94.4 µm) to over 400 µm (434 µm) (Figure 18a). Morphology of the coating across its cross-section (Figure 18d) suggested a diverse growth of crystallites in individual micro-areas. The analysis of chemical composition across the coating section (Figure 19 and Figure 20) demonstrated considerable changes, particularly for concentration of Cl, which could locally reach high values (Figure 20b). Chlorine was also the element, which occurred along with the zinc coating (Figure 20c,g).

The comparison of the thickness of zinc coating on steel in concrete without chlorides (Figure 15) and with chlorides (Figure 18) (though they are different specimens) indicated the presence of chlorides significantly reduced its thickness. That phenomenon was expected as a high concentration of chlorides always causes the corrosion of zinc coating [33]. The analysis of microscopic images showed the zinc coating at any place was completely damaged. Hence, high densities of corrosion current recorded during tests were always related to the dissolution of zinc, and not reinforcing steel protected by zinc. Corrosion products of zinc, with a smaller volume than those of iron, also did not cause cracks in the concrete cover of the reinforcement. Apparently, corrosion products of zinc filling the porous structure of concrete in the interfacial zone had enough space, and no undesirable critical values of tensile stress in concrete were triggered.

## 6. Discussion

The analysis of changes in a function of time of corrosion potential *E_corr_* of steel reinforcement in concrete very clearly demonstrated (Figure 4) that corrosion potential of black reinforcing steel, both in case of concrete without additives (group *B*), and with chlorides (group *B-Cl*) was relatively close within the range of −0.1–−0.5 V. While evaluating obtained results with reference to standard criteria [53] for testing potential of the reinforcement in reinforced concrete structures, the information about the corrosion probability of 50%–95% was obtained after calculating results per the potential of copper-sulphate electrode. It should be pointed out that the lowest values of potential up to the level of −0.45 V were observed for concrete with chlorides during the first weeks of testing. And distributions of corrosion potential of galvanized reinforcing steel in line with expectations were characterized by lower values which directly resulted from zinc position in the electrochemical series of metals. In concrete without chlorides, zinc as less noble metal than iron, being the main component of tested low-carbon reinforcing steel, had the potential of ca. 0.3–0.5 V lower than the potential of black steel when in direct contact with concrete pore solution. For concrete with chlorides, the measured corrosion potential of galvanized reinforcement demonstrated the similar difference in potentials. However, the spread of potential values for the specimens from the group *G-Cl* was considerably greater than for the group *B-Cl*.

**Figure 21:**
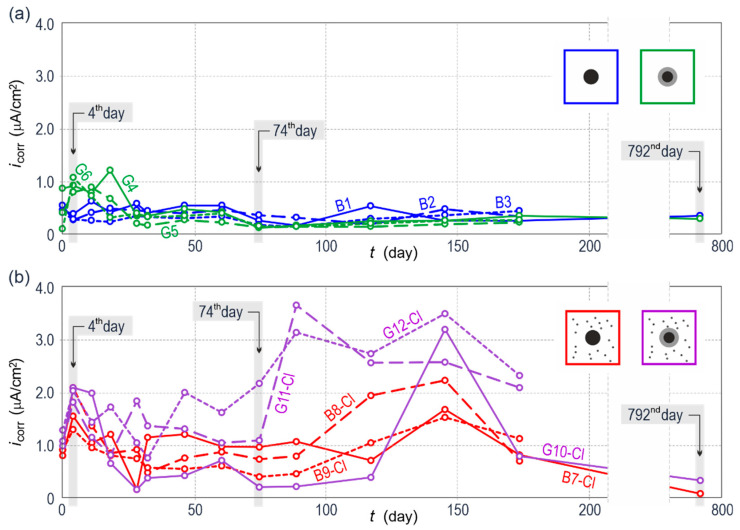
Changes in the function of time for corrosion current densities *i*_corr_ of galvanized and black steel reinforcement (**a**) in concrete without chlorides, (**b**) in concrete with chlorides; selected measuring series presented as diagrams and analyzed in details as part of EIS and LPR measurements are marked with grey color.

From a scientific perspective, the analysis of results from electrochemical tests (particularly density of corrosion current) seems to be very interesting during a 2-year period of performed tests. Figure 21 presents the comparison of changes in corrosion current densities *i*_corr_ within two years of measurements, obtained on the basis of analyses of polarization curves of reinforcing steel in the concrete specimens. Results for galvanized steel (*G*) and black steel (*B*) in concrete without additives are shown in Figure 21a, whereas results for similar rebars in concrete with chlorides are illustrated in Figure 21b.

As shown in Figure 21a, black reinforcing steel in concrete without additives (*B*) generally had stable but the low density of corrosion current through the whole period of measurements, which in accordance with criteria for evaluating corrosion risk to steel in concrete, could indicate the passivation. Microscopic tests (Figure 12b) demonstrated the presence of oxygen on the surface of steel rebar which probably indicated the presence of a passive layer of oxides that is characteristic for highly alkaline concrete pore solution (pH > 12.5).

In case of galvanized steel reinforcement in concrete without chlorides (*G*), elevated values of corrosion current density (oscillating around 1 µA/cm^2^) were observed in the initial phase of measurements (until day 18 of measurements). Then, corrosion current density stabilized at the level of 0.3–0.5 µA/cm^2^, which was a slightly better result than for black steel. The already mentioned quite a rapid increase in corrosion rate for galvanized steel in concrete without chloride content was related to zinc corrosion in highly alkaline concrete pore solution. At pH > 13.3 corrosion of galvanized coat on reinforcement was initiated.

Considering galvanized and black steel in concrete with chlorides, dynamics of changes in corrosion current densities and spread of those values during two-year measurements was very diversified (Figure 21b). During first 74 days of measurements, density of corrosion current for black steel did not exceed the level of 1.3 µA/cm^2^, whereas for galvanized steel values of *i*_corr_ were higher, even up to 2 µA/cm^2^. After day 74, a further increase in corrosion current densities was observed for both galvanized and black steel. However, a wider spread of values was found for reinforcing steel protected with the coating. Within that period, values of corrosion current density of the specimens with galvanized steel reached very high values of 3.8 µA/cm^2^. According to microscopic tests, zinc was found to be the only corrodible metal (Figure 20) on the undamaged structure of steel matrix which, considering the operational safety of reinforced concrete structures, is very important and positive information.

To sum it up, there was very strong corrosion of the reinforcement within the whole discussed period of two years of electrochemical testing according to criteria [51] for evaluating corrosion risk to reinforcing steel. Interestingly, after an 18-month interval in measurements determined densities of corrosion current dramatically decreased on day 792 of measurements. Corrosion of black steel was just inhibited as an insulating barrier from corrosion products of iron was formed. The microscopic tests (Figure 13) showed that a layer of corrosion products was thick enough to hinder diffusion of oxygen required for corrosion development. Diffusion-controlled electrochemical processes were also confirmed by the Nyquist plot (Figure 8a). It should be pointed out that the specimens were kept under rather stable thermal and humid conditions (temperature 20 ± 2 °C and relative humidity 50 ± 10%) during a very long, 18-month interval in measurements.

It is worth mentioning that despite an intensive development of corrosion on black steel in concrete with chlorides, no cracks were found on concrete surface of the test elements. It could be explained by a small diameter of rebars with reference to the thickness of the concrete cover. Thus, the volume of accumulating corrosion products did not exceed the available volume of concrete pores adjacent to rebars. Consequently, no critical values of tensile stress in concrete were observed.

## 7. Conclusions

The following conclusions can be drawn from two-year comparative tests on efficiency of the hot-dip galvanized zinc coating applied on reinforcing steel B500SP in concrete.

- Comparative tests of changes in the density of corrosion current in the concrete specimens without chlorides basically demonstrated no development of corrosion, and possible passivation was expected in case of black steel. Higher densities of corrosion current were observed for galvanized steel during the first days of testing. The reason was the dissolution of zinc after the contact with initially high pH of concrete pore solution.
- Six-month measurements demonstrated a higher density of corrosion current in the concrete specimens with high 3% concentration of chlorides, which unambiguously indicated corrosion in concrete reinforced with galvanized or black steel. Microscopic observations indicated zinc was the only metal corroding with the reinforcement made of galvanized steel, while the rebar surface showed no signs of corrosion.
- Densities of corrosion current determined for selected specimens dramatically decreased after an 18-month interval in measurements. Corrosion was inhibited on black steel as an insulating barrier of corrosion products was formed. That result was also confirmed with microscopic observations.
- The analysis of structural bonds in steel without or with zinc coating, containing concrete without or with chlorides demonstrated that the coating formed during hot dip galvanizing had varied thickness measured in the circumference at the cross-section of the rebar where the thickest layer of zinc was always observed at oblique ribs of the rebar.
- Additionally, the structural tests showed that the chemical composition at the section of the zinc coating was very homogeneous, and the layer of corrosion products indicated an increased content of oxygen, particularly for zinc coating in concrete with chlorides.
- Results obtained from corrosion (LPR, EIS) and structural (SEM, EDS) tests on the specimens of concrete reinforced with steel B500SP demonstrated a very favorable impact of zinc coating on rebars by providing effective protection against corrosion in chloride environment.

## Appendix A

**Table A1:** Comparison of results from analyzing polarization curves of reinforcing steel in concrete, obtained for three selected measuring series on day 4, 74, and 792.

Specimen No.	Δ*t*	*E_Ag|AgCl_*	*b_a_*	*b_c_*	*B*	*R_p_*	*R_p_A*	*i*_corr_
	(day)	(V)	(mV)	(mV)	(V)	(kΩ)	(kΩcm^2^)	(µA/cm^2^)
*B1*	4	−0.300	176.8	122.5	0.03142	2.647	80.48	0.39
*B2*	4	−0.290	180.3	73.5	0.02267	2.719	82.65	0.27
*B3*	4	−0.261	187.4	82.6	0.02489	2.924	88.90	0.28
*G4*	4	−0.722	267.3	167.9	0.04477	1.598	48.58	0.92
*G5*	4	−0.735	159.0	205.9	0.03896	1.609	48.92	0.80
*G6*	4	−0.726	153.6	182.3	0.03620	1.108	33.67	1.08
*B7-Cl*	4	−0.437	167.5	128.0	0.03151	0.668	20.31	1.55
*B8-Cl*	4	−0.460	166.8	155.3	0.03492	0.552	16.78	2.08
*B9-Cl*	4	−0.452	161.9	115.4	0.02926	0.738	22.45	1.30
*G10-Cl*	4	−0.919	177.9	112.4	0.02991	0.472	14.34	2.09
*G11-Cl*	4	−0.905	177.6	103.1	0.02833	0.513	15.59	1.82
*G12-Cl*	4	−0.906	217.9	177.1	0.04242	0.690	20.97	2.02
*B1*	74	−0.411	28.8	49.8	0.00793	1.010	30.71	0.26
*B2*	74	−0.393	41.1	48.7	0.00969	0.866	26.33	0.37
*B3*	74	−0.404	42.6	49.4	0.00993	1.841	55.97	0.18
*G4*	74	−0.845	21.9	71.7	0.00728	2.001	60.84	0.12
*G5*	74	−0.844	40.1	63.2	0.01065	2.582	78.49	0.14
*G6*	74	−0.867	35.1	70.9	0.01020	2.161	65.69	0.16
*B7-Cl*	74	−0.390	50.7	52.8	0.01123	0.380	11.55	0.97
*B8-Cl*	74	−0.362	48.9	59.6	0.01166	0.517	15.73	0.74
*B9-Cl*	74	−0.357	39.4	58.4	0.01021	0.811	24.65	0.41
*G10-Cl*	74	−0.664	34.6	40.5	0.00810	1.209	36.75	0.22
*G11-Cl*	74	−0.835	176.9	84.7	0.02486	0.747	22.70	1.09
*G12-Cl*	74	−0.870	69.3	61.6	0.01417	0.215	6.54	2.17
*B1*	792	−0.240	77.9	68.2	0.01580	1.477	44.90	0.35
*G4*	792	−0.851	53.2	90.6	0.01456	1.646	50.04	0.29
*B7-Cl*	792	−0.040	58.6	45.1	0.01107	55.292	1680.87	0.01
*G10-Cl*	792	−0.806	51.9	56.0	0.01169	1.121	34.07	0.34

**Table A2:** Comparison of results from analyzing impedance spectra of reinforcing steel in concrete, obtained for three selected measuring series on day 4, 74, and 792.

Specimen No.	Δ*t*	*E_Ag|AgCl_*	*R_c_*	*R_f_*	*CPE_f_*		*R_t_*	*CPE*_0_		*i*_corr_
					*Y_f_*	*α_f_*		*Y*_0_	*α*_0_	
	(day)	(V)	(Ω)	(Ω)	(Fs^α−1^)	-	(Ω)	(Fs^α−1^)	-	(µA/cm^2^)
*B1*	4	−0.305	415	–	–	–	3388	2.31 × 10^−3^	0.597	0.31
*B2*	4	−0.289	234	–	–	–	3634	2.16 × 10^−3^	0.615	0.21
*B3*	4	−0.265	250	–	–	–	5925	1.98 × 10^−3^	0.622	0.14
*G4*	4	−0.721	317	–	–	–	2181	1.89 × 10^−3^	0.660	0.68
*G5*	4	−0.732	384	–	–	–	2324	1.76 × 10^−3^	0.625	0.55
*G6*	4	−0.728	247	–	–	–	1618	2.02 × 10^−3^	0.661	0.74
*B7-Cl*	4	−0.437	253	–	–	–	468	3.52 × 10^−3^	0.539	2.21
*B8-Cl*	4	−0.458	294	–	–	–	376	3.98 × 10^−3^	0.529	3.06
*B9-Cl*	4	−0.451	415	–	–	–	503	4.77 × 10^−3^	0.528	1.91
*G10-Cl*	4	−0.915	216	75	2.12 × 10^−3^	0.448	333	6.24 × 10^−2^	0.641	2.96
*G11-Cl*	4	−0.902	228	95	2.71 × 10^−3^	0.431	371	3.49 × 10^−2^	0.643	2.51
*G12-Cl*	4	−0.905	276	138	1.90 × 10^−3^	0.424	539	3.03 × 10^−2^	0.648	2.59
*B1*	74	−0.406	548	–	–	–	857	2.72 × 10^−3^	0.708	0.30
*B2*	74	−0.392	567	–	–	–	1388	2.63 × 10^−3^	0.647	0.23
*B3*	74	−0.405	554	–	–	–	1565	2.40 × 10^−3^	0.681	0.21
*G4*	74	−0.846	539	–	–	–	1430	1.86 × 10^−3^	0.673	0.17
*G5*	74	−0.846	613	–	–	–	2863	1.48 × 10^−3^	0.631	0.12
*G6*	74	−0.870	514	–	–	–	2097	1.39 × 10^−3^	0.608	0.16
*B7-Cl*	74	−0.378	449	–	–	–	423	4.53 × 10^−3^	0.399	0.87
*B8-Cl*	74	−0.349	708	–	–	–	417	3.50 × 10^−3^	0.456	0.92
*B9-Cl*	74	−0.348	849	–	–	–	684	2.36 × 10^−3^	0.456	0.49
*G10-Cl*	74	−0.663	890	382	6.14 × 10^−4^	0.447	1007	6.52 × 10^−3^	0.618	0.26
*G11-Cl*	74	−0.831	477	175	6.71 × 10^−4^	0.426	465	5.32 × 10^−2^	0.637	1.76
*G12-Cl*	74	−0.865	419	151	8.49 × 10^−4^	0.441	488	5.57 × 10^−2^	0.612	0.95
*B1*	792	−0.199	1904	–	–	–	1262	7.69 × 10^−4^	0.346	0.41
*G4*	792	−0.833	630	–	–	–	1413	2.19 × 10^−4^	0.462	0.34
*B7-Cl*	792	−0.052	30,440	18,880	7.00 × 10^−6^	0.420	735,900	6.54 × 10^−5^	0.498	0.00
*G10-Cl*	792	−0.806	2620	–	–	–	1222	3.67 × 10^−4^	0.421	0.31
